# Crystal structure of 5,7-diphenyl-4,7-di­hydro­tetra­zolo[1,5-*a*]pyrimidine

**DOI:** 10.1107/S2056989015002984

**Published:** 2015-02-21

**Authors:** Ivy K. Price, Celine Rougeot, Jason E. Hein

**Affiliations:** a Chemistry and Chemical Biology, University of California, Merced, 5200 North Lake Road, Merced, CA 95343, USA

**Keywords:** crystal structure, tetra­zolo[1,5-*a*]pyrimidine, hydrogen bonding

## Abstract

In the title mol­ecule, C_16_H_13_N_5_, the plane of the tetra­zole ring forms dihedral angles of 16.37 (7) and 76.59 (7)° with the two phenyl rings. The dihedral angle between the phenyl rings is 68.05 (6)°. The pyrimidine ring is in a flattened boat conformation. In the crystal, mol­ecules are linked by pairs of N—H⋯N hydrogen bonds, forming inversion dimers.

## Related literature   

For the synthesis, see: Desenko *et al.* (2001 ▸); Ghorbani-Vaghei *et al.* (2013 ▸).
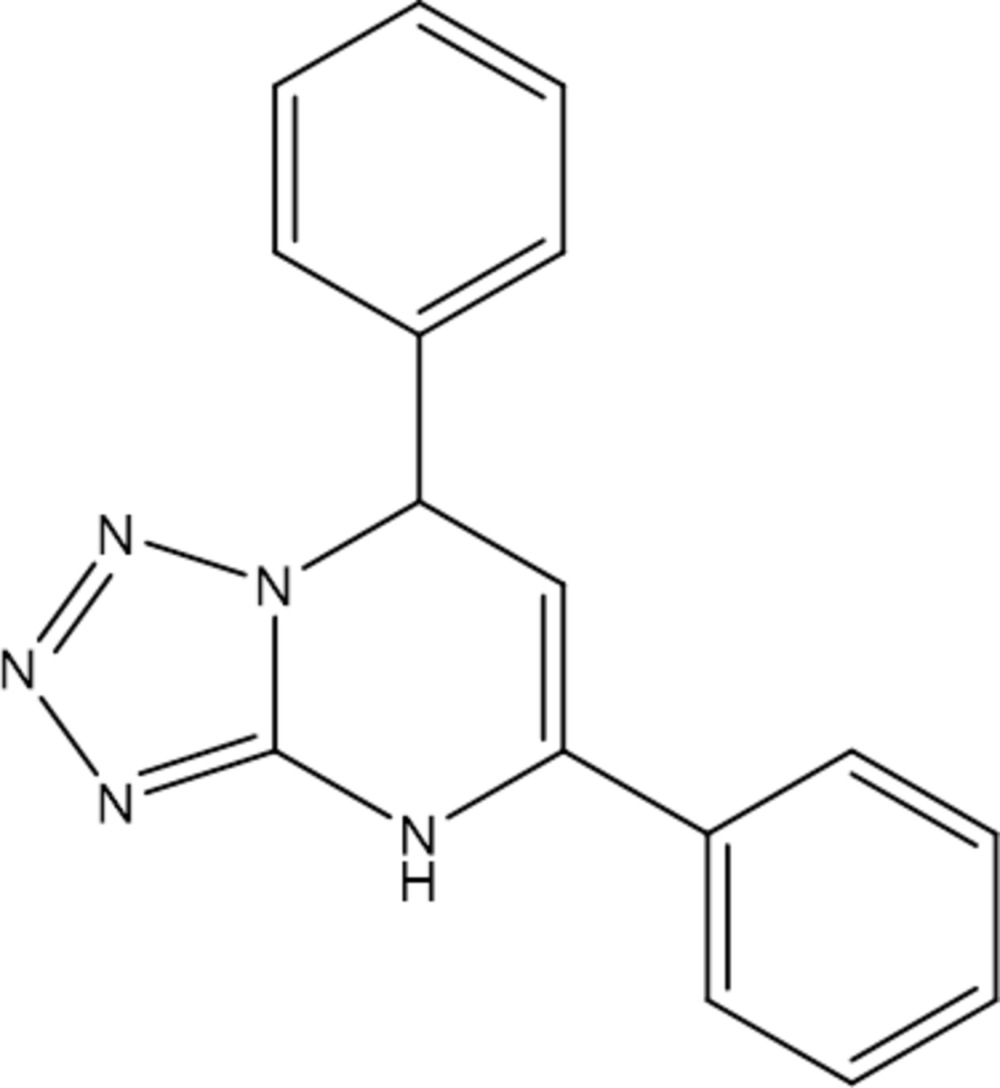


## Experimental   

### Crystal data  

C_16_H_13_N_5_
*M* *_r_* = 275.31
Orthorhombic, 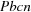

*a* = 12.6931 (8) Å
*b* = 10.9284 (6) Å
*c* = 18.8915 (12) Å
*V* = 2620.5 (3) Å^3^
*Z* = 8
Cu *K*α radiation
μ = 0.71 mm^−1^
*T* = 100 K
0.28 × 0.22 × 0.15 mm

### Data collection  

Bruker D8 APEX Cu diffractometer
Absorption correction: multi-scan (*SADABS*; Bruker, 2012 ▸) *T*_min_ = 0.631, *T*_max_ = 0.753
13662 measured reflections
2357 independent reflections
2047 reflections with *I* > 2σ(*I*)
*R*_int_ = 0.048

### Refinement  

*R*[*F* ^2^ > 2σ(*F* ^2^)] = 0.035
*wR*(*F* ^2^) = 0.093
*S* = 1.06
2357 reflections
194 parameters
H atoms treated by a mixture of independent and constrained refinement
Δρ_max_ = 0.13 e Å^−3^
Δρ_min_ = −0.23 e Å^−3^

### 

Data collection: *APEX2* (Bruker, 2013 ▸); cell refinement: *SAINT* (Bruker, 2013 ▸); data reduction: *SAINT*; program(s) used to solve structure: *SHELXS97* (Sheldrick, 2008 ▸); program(s) used to refine structure: *SHELXL2013* (Sheldrick, 2015 ▸); molecular graphics: *OLEX2* (Dolomanov *et al.*, 2009 ▸); software used to prepare material for publication: *OLEX2*.

## Supplementary Material

Crystal structure: contains datablock(s) I. DOI: 10.1107/S2056989015002984/lh5746sup1.cif

Structure factors: contains datablock(s) I. DOI: 10.1107/S2056989015002984/lh5746Isup2.hkl

Supporting information file. DOI: 10.1107/S2056989015002984/lh5746Isup3.cdx

Supporting information file. DOI: 10.1107/S2056989015002984/lh5746Isup4.cml

**Fig. 1.**
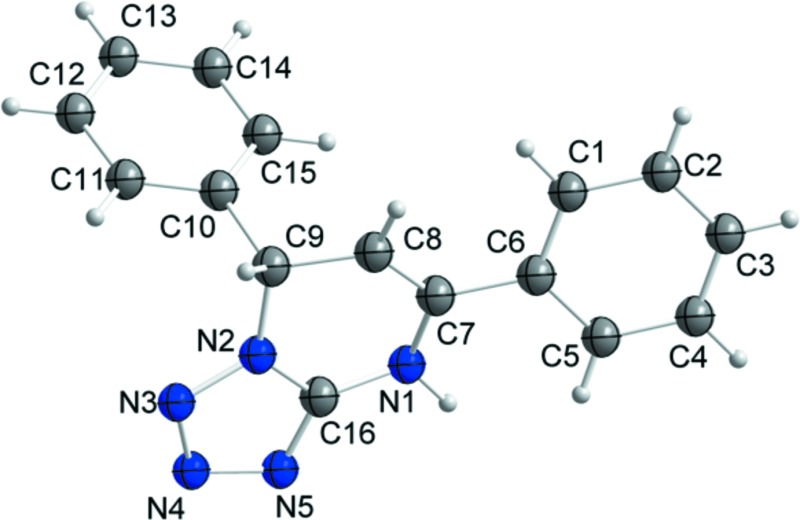
The molecular structure of the title compound, showing 50% probability displacement ellipsoids.

. DOI: 10.1107/S2056989015002984/lh5746fig1.tif
The mol­ecular structure of the title compound, showing 50% probability displacement ellipsoids.

**Fig. 2.**
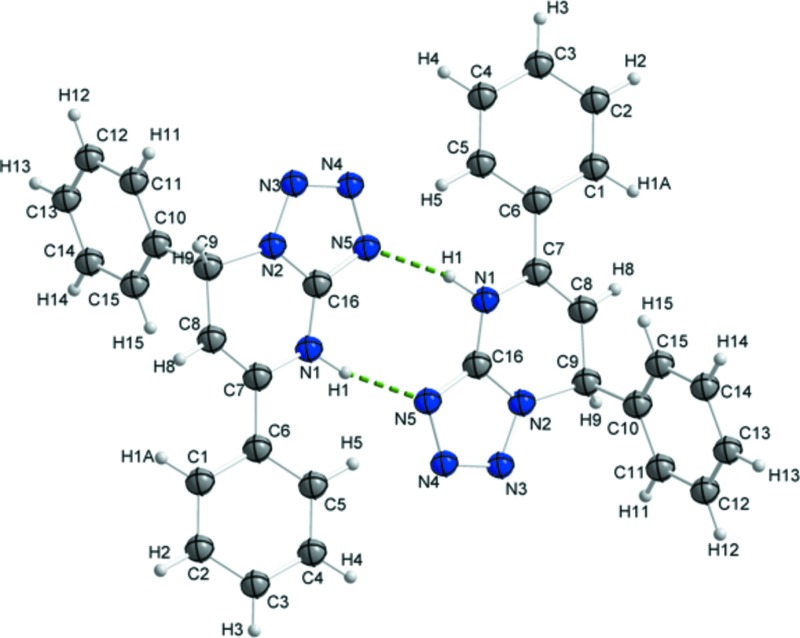
A pair of molecules linked by intermolecular N—H···N hydrogen bonds (dashed lines).

. DOI: 10.1107/S2056989015002984/lh5746fig2.tif
A pair of mol­ecules linked by inter­molecular N—H⋯N hydrogen bonds (dashed lines).

CCDC reference: 1048926

Additional supporting information:  crystallographic information; 3D view; checkCIF report

## Figures and Tables

**Table 1:** Hydrogen-bond geometry (, )

*D*H*A*	*D*H	H*A*	*D* *A*	*D*H*A*
N1H1N5^i^	0.94(2)	1.99(2)	2.908(2)	165(1)

Symmetry code: (i) 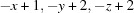.

## supplementary crystallographic information

### S1. Comment

The title compound was synthesized via condensation between chalcone and aminotetrazole using a literature procedure to produce a racemic mixture (Desenko *et al.*, 2001; Ghorbani-Vaghei *et al.*, 2013). Successive recrystallization of this compound from a variety of solvents identified multiple crystal isoforms, which appear to be metastable and rearrange to give the crystal structure reported herein.

The molecular structure of the title compound is shown in Fig. 1. The tetrazole ring [N2–N5/C16] forms dihedral angles of 16.37 (7) [C1–C6] and 76.59 (7)° [C10–C15] with the two phenyl rings. The dihedral angle between the phenyl rings is 68.05 (6)°. The pyrimidine ring [N1/N2/C7/C8/C9/C16] is in a flattened boat conformation with N1 and C9 deviating by 0.1222 (10) and 0.2478 (13) Å respectively, from the mean plane of the other four atoms [N2/C7/C8/C16]. In the crystal, pairs of molecules are linked by N—H···N hydrogen bonds to form invesion dimers.

### S2. Experimental

1H-Tetrazol-5-amine (2.0g, 23.51mmol) and (E)-chalcone (5.39g, 25.9mmol) was added to DMF (3.92ml) in an oven dried vial then stirred overnight at 423K. Then while still heating, the product was diluted with toluene (0.4mL) and stirred for 2 more hours. The solid precipitate was collected via vacuum filtration, rinsed with toluene and placed on high vacuum until dry. 4.34g of white solid was collected. X-ray quality crystals were grown from slow evaporation of a solution of the title compound in dichloromethane.

### S3. Refinement

H atoms bonded to C atoms were included in calculated positions with C—H = 0.95Å and U_iso_(H) = 1.2U_eq_(C). The H atom bonded to N was refined independently with an isotropic displacement parameter.

### Crystal data

**Table:**

C_16_H_13_N_5_	*D*_x_ = 1.396 Mg m^−^^3^
*M**_r_* = 275.31	Cu *K*α radiation, λ = 1.54178 Å
Orthorhombic, *P**b**c**n*	Cell parameters from 6133 reflections
*a* = 12.6931 (8) Å	θ = 4.2–68.2°
*b* = 10.9284 (6) Å	µ = 0.71 mm^−^^1^
*c* = 18.8915 (12) Å	*T* = 100 K
*V* = 2620.5 (3) Å^3^	Block, colorless
*Z* = 8	0.28 × 0.22 × 0.15 mm
*F*(000) = 1152	

### Data collection

**Table:**

Bruker D8 APEX Cu diffractometer	2357 independent reflections
Radiation source: Micro Focus Rotating Anode, Bruker FR-591	2047 reflections with *I* > 2σ(*I*)
Multilayer Mirrors monochromator	*R*_int_ = 0.048
Detector resolution: 8.0 pixels mm^-1^	θ_max_ = 68.2°, θ_min_ = 4.7°
ω and φ scans	*h* = −10→15
Absorption correction: multi-scan (*SADABS*; Bruker, 2012)	*k* = −13→11
*T*_min_ = 0.631, *T*_max_ = 0.753	*l* = −22→15
13662 measured reflections	

### Refinement

**Table:**

Refinement on *F*^2^	Primary atom site location: structure-invariant direct methods
Least-squares matrix: full	Secondary atom site location: difference Fourier map
*R*[*F*^2^ > 2σ(*F*^2^)] = 0.035	Hydrogen site location: mixed
*wR*(*F*^2^) = 0.093	H atoms treated by a mixture of independent and constrained refinement
*S* = 1.06	*w* = 1/[σ^2^(*F*_o_^2^) + (0.0481*P*)^2^ + 0.7255*P*] where *P* = (*F*_o_^2^ + 2*F*_c_^2^)/3
2357 reflections	(Δ/σ)_max_ = 0.001
194 parameters	Δρ_max_ = 0.13 e Å^−^^3^
0 restraints	Δρ_min_ = −0.23 e Å^−^^3^

### Special details

**Table:**

Experimental. Absortion correction: SADABS-2012/1 (Bruker,2012) was used for absorption correction. wR2(int) was 0.0847 before and 0.0589 after correction. The Ratio of minimum to maximum transmission is 0.8385. The λ/2 correction factor is 0.0015.
Geometry. All e.s.d.'s (except the e.s.d. in the dihedral angle between two l.s. planes) are estimated using the full covariance matrix. The cell e.s.d.'s are taken into account individually in the estimation of e.s.d.'s in distances, angles and torsion angles; correlations between e.s.d.'s in cell parameters are only used when they are defined by crystal symmetry. An approximate (isotropic) treatment of cell e.s.d.'s is used for estimating e.s.d.'s involving l.s. planes.

### Fractional atomic coordinates and isotropic or equivalent isotropic displacement parameters (Å^2^)

**Table:**

	*x*	*y*	*z*	*U*_iso_*/*U*_eq_	
N5	0.47790 (8)	0.87666 (9)	1.05756 (5)	0.0178 (2)	
N4	0.47318 (8)	0.77893 (9)	1.10314 (6)	0.0196 (3)	
N3	0.39481 (9)	0.70757 (10)	1.08856 (6)	0.0206 (3)	
N2	0.34601 (8)	0.75851 (10)	1.03147 (6)	0.0175 (3)	
N1	0.36681 (8)	0.92953 (10)	0.95763 (5)	0.0174 (2)	
H1	0.4132 (13)	0.9922 (16)	0.9444 (8)	0.034 (4)*	
C16	0.39775 (10)	0.86051 (11)	1.01341 (6)	0.0158 (3)	
C9	0.24638 (10)	0.71622 (12)	1.00051 (7)	0.0184 (3)	
H9	0.2508	0.6259	0.9925	0.022*	
C8	0.23680 (10)	0.77919 (12)	0.93004 (7)	0.0191 (3)	
H8	0.1897	0.7452	0.8963	0.023*	
C7	0.29038 (10)	0.88019 (11)	0.91166 (6)	0.0170 (3)	
C10	0.15611 (10)	0.74218 (12)	1.05162 (7)	0.0188 (3)	
C11	0.13730 (11)	0.66105 (12)	1.10670 (7)	0.0222 (3)	
H11	0.1786	0.5889	1.1109	0.027*	
C12	0.05843 (11)	0.68479 (13)	1.15568 (7)	0.0263 (3)	
H12	0.0466	0.6294	1.1936	0.032*	
C13	−0.00323 (11)	0.78893 (13)	1.14952 (7)	0.0263 (3)	
H13	−0.0579	0.8045	1.1827	0.032*	
C14	0.01545 (11)	0.87026 (13)	1.09460 (7)	0.0258 (3)	
H14	−0.0264	0.9420	1.0903	0.031*	
C15	0.09509 (10)	0.84739 (12)	1.04586 (7)	0.0218 (3)	
H15	0.1079	0.9037	1.0086	0.026*	
C6	0.27368 (10)	0.94608 (11)	0.84397 (6)	0.0181 (3)	
C1	0.18077 (11)	0.92813 (12)	0.80523 (7)	0.0226 (3)	
H1A	0.1285	0.8737	0.8227	0.027*	
C2	0.16440 (11)	0.98872 (13)	0.74191 (7)	0.0266 (3)	
H2	0.1018	0.9741	0.7157	0.032*	
C3	0.23888 (11)	1.07083 (13)	0.71640 (7)	0.0255 (3)	
H3	0.2267	1.1137	0.6734	0.031*	
C4	0.33095 (11)	1.08969 (12)	0.75403 (7)	0.0238 (3)	
H4	0.3823	1.1454	0.7367	0.029*	
C5	0.34843 (10)	1.02735 (11)	0.81705 (7)	0.0206 (3)	
H5	0.4123	1.0403	0.8422	0.025*	

### Atomic displacement parameters (Å^2^)

**Table:**

	*U*^11^	*U*^22^	*U*^33^	*U*^12^	*U*^13^	*U*^23^
N5	0.0175 (5)	0.0196 (5)	0.0163 (5)	0.0002 (4)	−0.0011 (4)	0.0016 (4)
N4	0.0181 (5)	0.0212 (5)	0.0195 (5)	−0.0002 (4)	−0.0005 (4)	0.0035 (5)
N3	0.0184 (5)	0.0232 (5)	0.0203 (6)	−0.0003 (4)	−0.0025 (5)	0.0041 (5)
N2	0.0169 (5)	0.0183 (5)	0.0173 (5)	−0.0012 (4)	−0.0019 (4)	0.0020 (4)
N1	0.0186 (5)	0.0177 (5)	0.0159 (5)	−0.0024 (5)	−0.0029 (4)	0.0010 (4)
C16	0.0155 (6)	0.0160 (6)	0.0159 (6)	0.0006 (5)	0.0008 (5)	−0.0013 (5)
C9	0.0177 (6)	0.0185 (6)	0.0191 (6)	−0.0036 (5)	−0.0016 (5)	−0.0018 (5)
C8	0.0174 (6)	0.0223 (6)	0.0177 (6)	−0.0020 (5)	−0.0017 (5)	−0.0024 (5)
C7	0.0151 (6)	0.0205 (6)	0.0154 (6)	0.0021 (5)	−0.0004 (5)	−0.0036 (5)
C10	0.0182 (6)	0.0214 (6)	0.0168 (6)	−0.0056 (5)	−0.0032 (5)	−0.0023 (5)
C11	0.0233 (7)	0.0219 (6)	0.0213 (7)	−0.0047 (6)	−0.0011 (5)	−0.0003 (6)
C12	0.0286 (7)	0.0301 (7)	0.0203 (7)	−0.0105 (6)	0.0014 (6)	0.0002 (6)
C13	0.0201 (7)	0.0368 (8)	0.0219 (7)	−0.0059 (6)	0.0021 (6)	−0.0082 (6)
C14	0.0216 (7)	0.0313 (7)	0.0244 (7)	0.0018 (6)	−0.0035 (6)	−0.0050 (6)
C15	0.0209 (7)	0.0246 (7)	0.0198 (6)	−0.0014 (6)	−0.0029 (5)	0.0004 (6)
C6	0.0192 (6)	0.0187 (6)	0.0162 (6)	0.0035 (5)	−0.0001 (5)	−0.0029 (5)
C1	0.0199 (7)	0.0252 (7)	0.0226 (7)	0.0008 (6)	−0.0013 (6)	0.0004 (6)
C2	0.0242 (7)	0.0329 (7)	0.0227 (7)	0.0052 (6)	−0.0057 (6)	−0.0013 (6)
C3	0.0318 (8)	0.0266 (7)	0.0181 (6)	0.0079 (6)	−0.0015 (6)	0.0023 (6)
C4	0.0291 (7)	0.0227 (6)	0.0197 (6)	0.0005 (6)	0.0011 (6)	0.0007 (6)
C5	0.0219 (7)	0.0212 (6)	0.0188 (6)	0.0000 (5)	−0.0022 (5)	−0.0017 (6)

### Geometric parameters (Å, º)

**Table:**

N5—N4	1.3732 (15)	C12—H12	0.9500
N5—C16	1.3274 (16)	C12—C13	1.386 (2)
N4—N3	1.2937 (16)	C13—H13	0.9500
N3—N2	1.3627 (15)	C13—C14	1.387 (2)
N2—C16	1.3381 (16)	C14—H14	0.9500
N2—C9	1.4680 (16)	C14—C15	1.3899 (19)
N1—H1	0.937 (18)	C15—H15	0.9500
N1—C16	1.3541 (16)	C6—C1	1.4018 (18)
N1—C7	1.4093 (16)	C6—C5	1.3955 (18)
C9—H9	1.0000	C1—H1A	0.9500
C9—C8	1.5035 (18)	C1—C2	1.3831 (19)
C9—C10	1.5250 (18)	C2—H2	0.9500
C8—H8	0.9500	C2—C3	1.390 (2)
C8—C7	1.3421 (18)	C3—H3	0.9500
C7—C6	1.4829 (17)	C3—C4	1.3832 (19)
C10—C11	1.3877 (19)	C4—H4	0.9500
C10—C15	1.3905 (19)	C4—C5	1.3896 (19)
C11—H11	0.9500	C5—H5	0.9500
C11—C12	1.3879 (19)		
			
C16—N5—N4	104.90 (10)	C11—C12—H12	119.8
N3—N4—N5	111.65 (10)	C13—C12—C11	120.32 (13)
N4—N3—N2	105.77 (10)	C13—C12—H12	119.8
N3—N2—C9	125.34 (10)	C12—C13—H13	120.2
C16—N2—N3	108.61 (10)	C12—C13—C14	119.51 (13)
C16—N2—C9	125.69 (11)	C14—C13—H13	120.2
C16—N1—H1	115.6 (10)	C13—C14—H14	119.8
C16—N1—C7	117.77 (10)	C13—C14—C15	120.31 (13)
C7—N1—H1	123.2 (10)	C15—C14—H14	119.8
N5—C16—N2	109.07 (11)	C10—C15—H15	119.9
N5—C16—N1	129.58 (11)	C14—C15—C10	120.14 (13)
N2—C16—N1	121.35 (11)	C14—C15—H15	119.9
N2—C9—H9	108.8	C1—C6—C7	120.16 (12)
N2—C9—C8	106.17 (10)	C5—C6—C7	121.75 (12)
N2—C9—C10	109.66 (10)	C5—C6—C1	118.09 (12)
C8—C9—H9	108.8	C6—C1—H1A	119.6
C8—C9—C10	114.50 (11)	C2—C1—C6	120.72 (13)
C10—C9—H9	108.8	C2—C1—H1A	119.6
C9—C8—H8	117.8	C1—C2—H2	119.8
C7—C8—C9	124.36 (12)	C1—C2—C3	120.46 (13)
C7—C8—H8	117.8	C3—C2—H2	119.8
N1—C7—C6	116.36 (11)	C2—C3—H3	120.2
C8—C7—N1	120.28 (11)	C4—C3—C2	119.52 (12)
C8—C7—C6	123.37 (12)	C4—C3—H3	120.2
C11—C10—C9	119.02 (12)	C3—C4—H4	119.9
C11—C10—C15	119.41 (12)	C3—C4—C5	120.14 (13)
C15—C10—C9	121.52 (12)	C5—C4—H4	119.9
C10—C11—H11	119.8	C6—C5—H5	119.5
C12—C11—C10	120.30 (13)	C4—C5—C6	121.04 (12)
C12—C11—H11	119.8	C4—C5—H5	119.5
			
N5—N4—N3—N2	0.30 (13)	C9—C10—C11—C12	177.42 (11)
N4—N5—C16—N2	0.52 (13)	C9—C10—C15—C14	−178.02 (12)
N4—N5—C16—N1	−179.19 (12)	C8—C9—C10—C11	158.67 (11)
N4—N3—N2—C16	0.03 (13)	C8—C9—C10—C15	−23.84 (17)
N4—N3—N2—C9	−173.39 (11)	C8—C7—C6—C1	18.98 (19)
N3—N2—C16—N5	−0.36 (14)	C8—C7—C6—C5	−161.31 (12)
N3—N2—C16—N1	179.38 (11)	C7—N1—C16—N5	168.75 (12)
N3—N2—C9—C8	−167.03 (11)	C7—N1—C16—N2	−10.94 (17)
N3—N2—C9—C10	68.78 (15)	C7—C6—C1—C2	−179.78 (12)
N2—C9—C8—C7	−19.03 (17)	C7—C6—C5—C4	−179.09 (12)
N2—C9—C10—C11	−82.14 (14)	C10—C9—C8—C7	102.10 (14)
N2—C9—C10—C15	95.35 (14)	C10—C11—C12—C13	0.9 (2)
N1—C7—C6—C1	−160.74 (11)	C11—C10—C15—C14	−0.54 (19)
N1—C7—C6—C5	18.97 (17)	C11—C12—C13—C14	−0.9 (2)
C16—N5—N4—N3	−0.52 (13)	C12—C13—C14—C15	0.3 (2)
C16—N2—C9—C8	20.66 (16)	C13—C14—C15—C10	0.5 (2)
C16—N2—C9—C10	−103.54 (14)	C15—C10—C11—C12	−0.12 (19)
C16—N1—C7—C8	12.02 (17)	C6—C1—C2—C3	−1.5 (2)
C16—N1—C7—C6	−168.25 (11)	C1—C6—C5—C4	0.62 (19)
C9—N2—C16—N5	173.03 (11)	C1—C2—C3—C4	1.4 (2)
C9—N2—C16—N1	−7.23 (19)	C2—C3—C4—C5	−0.3 (2)
C9—C8—C7—N1	4.52 (19)	C3—C4—C5—C6	−0.7 (2)
C9—C8—C7—C6	−175.19 (11)	C5—C6—C1—C2	0.50 (19)

### Hydrogen-bond geometry (Å, º)

**Table:**

*D*—H···*A*	*D*—H	H···*A*	*D*···*A*	*D*—H···*A*
N1—H1···N5^i^	0.937 (18)	1.992 (18)	2.9075 (15)	165.3 (14)

Symmetry code: (i) −*x*+1, −*y*+2, −*z*+2.
