# Supplementary figures and images for: Crystal structure of 5,7-diphenyl-4,7-di­hydro­tetra­zolo[1,5-a]pyrimidine
Source: Acta Crystallogr E Crystallogr Commun. 2015 Feb 21;71(Pt 3):o192. doi: 10.1107/S2056989015002984 (PMC4350716; doi:10.1107/S2056989015002984)

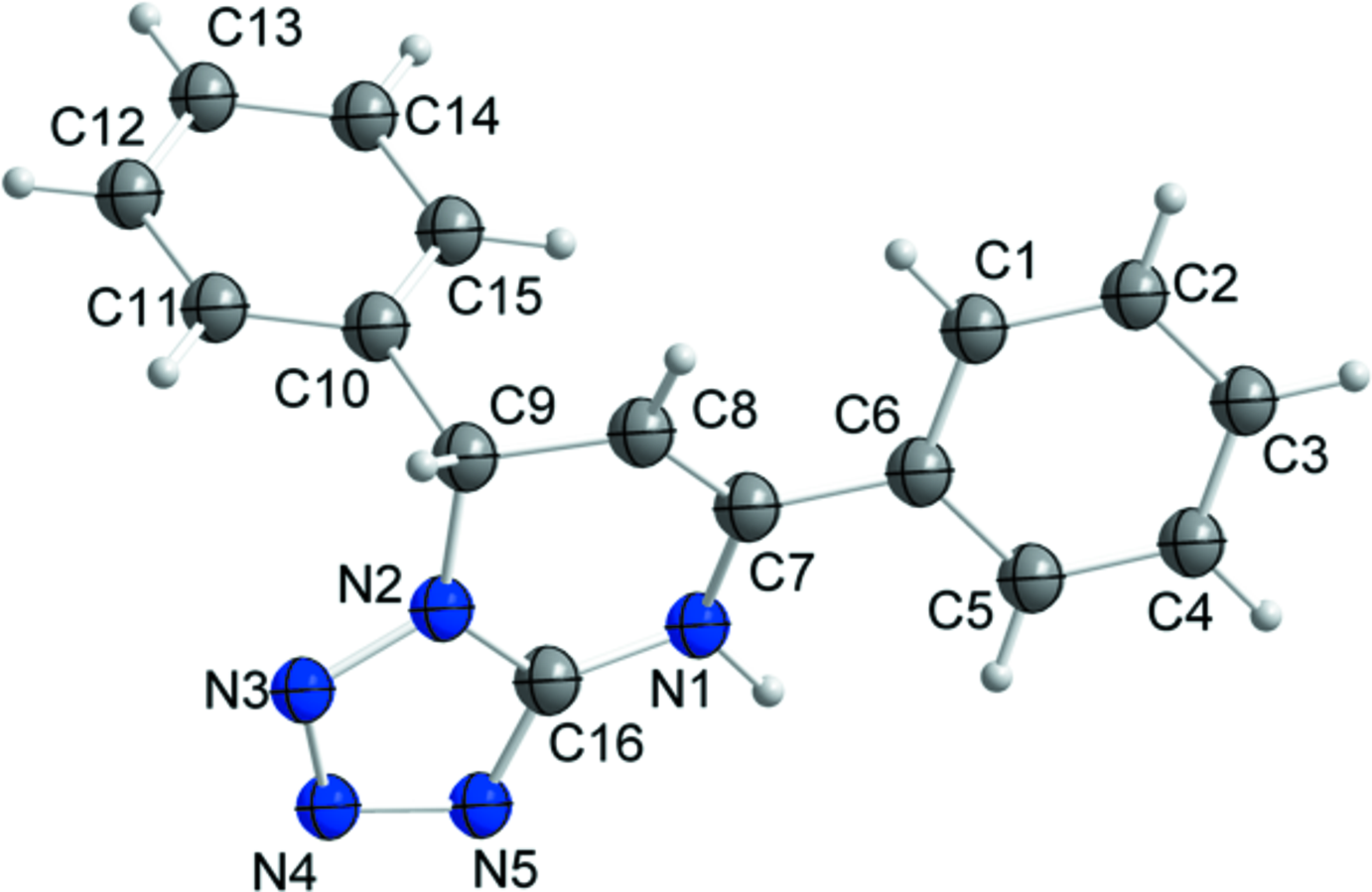

Supplement: Supplementary file 5 [file e-71-0o192-fig1.tif]

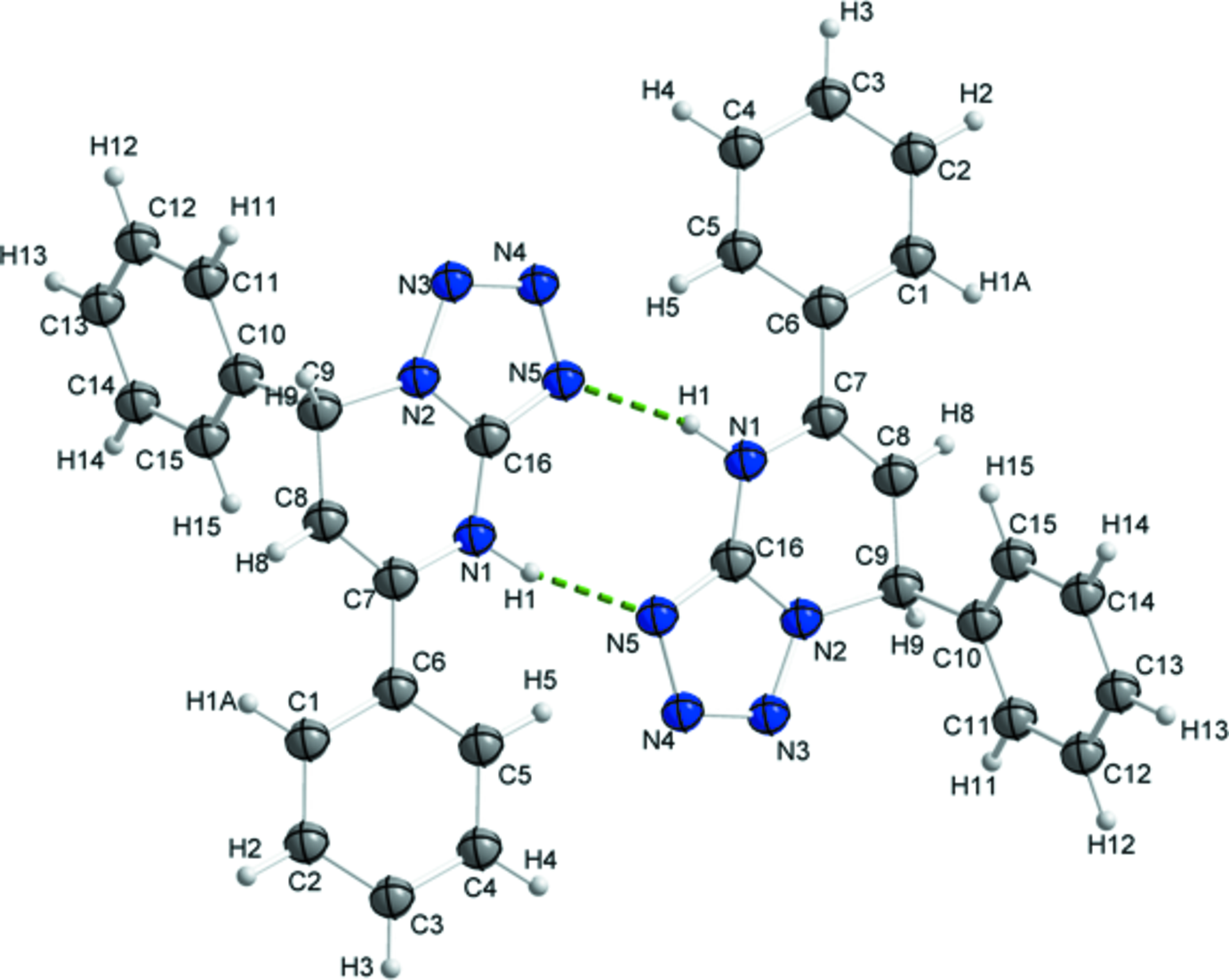

Supplement: Supplementary file 6 [file e-71-0o192-fig2.tif]
